# Novel Compounds Synergize With Venetoclax to Target KMT2A-Rearranged Pediatric Acute Myeloid Leukemia

**DOI:** 10.3389/fphar.2021.820191

**Published:** 2022-01-27

**Authors:** Claudia Tregnago, Maddalena Benetton, Ambra Da Ros, Giulia Borella, Giorgia Longo, Katia Polato, Samuela Francescato, Alessandra Biffi, Martina Pigazzi

**Affiliations:** Pediatric Haematology-Oncology and Hematopoietic Cell and Gene Therapy Division, Woman and Child Health Department, University-Hospital of Padova, Padova, Italy

**Keywords:** AML, KMT2A, venetoclax, synergy, targeted drugs

## Abstract

In pediatric acute myeloid leukemia (AML), fusions involving lysine methyltransferase 2A (KMT2A) are considered hallmarks of aggressive AML, for whom the development of targeted specific therapeutic agents to ameliorate classic chemotherapy and obtain a complete eradication of disease is urgent. In this study, we investigated the antiapoptotic proteins in a cohort of 66 pediatric AML patients, finding that 75% of the KMT2A-r are distributed in Q3 + Q4 quartiles of BCL-2 expression, and KMT2A-r have statistically significant high levels of BCL-2, phospho-BCL-2 S70, and MCL-1, indicating a high anti-apoptotic pathway activation. In an attempt to target it, we tested novel drug combinations of venetoclax, a B-cell lymphoma-2 (BCL-2) inhibitor, in KMT2A-MLLT3, for being the most recurrent, and KMT2A-AFDN, for mediating the worst prognosis, rearranged AML cell lines. Our screening revealed that both the bromodomain and extra-terminal domain (BET) inhibitor, I-BET151, and kinase inhibitor, sunitinib, decreased the BCL-2 family protein expression and significantly synergized with venetoclax, enhancing KMT2A-r AML cell line death. Blasts t (6; 11) KMT2A-AFDN rearranged, both from cell lines and primary samples, were shown to be significantly highly responsive to the combination of venetoclax and thioridazine, with the synergy being induced by a dramatic increase of mitochondrial depolarization that triggered blast apoptosis. Finally, the efficacy of novel combined drug treatments was confirmed in KMT2A-r AML cell lines or *ex vivo* primary KMT2A-r AML samples cultured in a three-dimensional system which mimics the bone marrow niche. Overall, this study identified that, by high-throughput screening, the most KMT2A-selective drugs converged in different but all mitochondrial apoptotic network activation, supporting the use of venetoclax in this AML setting. The novel drug combinations here unveiled provide a rationale for evaluating these combinations in preclinical studies to accelerate the introduction of targeted therapies for the life-threatening KMT2A-AML subgroup of pediatric AML.

## Introduction

Leukemia is the most common type of cancer in childhood, accounting for 25–30% of cancers in children and adolescents aged 0–18 years (Kuhlen et al., 2019).

Despite the refinement of risk stratification on the basis of clinical characteristics, molecular profiling, and detection of minimal residual disease after induction therapy, to date the overall survival for children with acute myeloid leukemia (AML) has not exceeded 70% (Pui et al., 2011; Pession et al., 2013; Elgarten and Aplenc, 2020). Collaborative international efforts have resulted in the convergence of treatment approaches for pediatric AML that, nevertheless, continue to be based primarily on intensive, conventional chemotherapy, followed by hematopoietic stem cell transplant for patients with high-risk AML or after experiencing a relapse (Zwaan et al., 2015).

In the last decades, data that emerged from sequencing and molecular profiling have identified molecular subsets with prognostic significance, with a pediatric AML molecular landscape that remains quite distinct from adults (Pigazzi et al., 2011; Masetti et al., 2013; Manara et al., 2014b; Manara et al., 2016; Bisio et al., 2017; Bolouri et al., 2018; Noort et al., 2018; Zampini et al., 2018). Among the recurrent lesions in pediatric patients, fusions involving lysine methyltransferase 2A (KMT2A, MLL) are considered hallmarks of aggressive AML, particularly in infants and early childhood. The 11q23-rearranged AML subgroup represents more than 20% of pediatric cases, with >80 recognized fusion partners, although the majority of leukemias result from KMT2A fusions with one of about six common partner genes, with KMT2A-MLLT3 being the most recurrent, and the prognostic significance is strictly dependent on the fusion partner, with the KMT2A-AFDN fusion associated with a particularly poor prognosis (Balgobind et al., 2009; Coenen et al., 2011; Pigazzi et al., 2011; Manara et al., 2014a; Meyer et al., 2018). This ever-expanding knowledge on leukemia biology is crucial to identify those molecular targets useful for the development of therapeutic agents to ameliorate classic chemotherapy and obtain complete eradication of the disease while reducing toxicity when possible (Mercher and Schwaller, 2019). In the adult AML field, there is a fervent and rapid evolution on the prognosis and management of the disease due to the Food and Drug Administration (FDA) approval of several new drugs targeting key molecular pathways involved in leukemia development, cell growth, and proliferation for different AML indications (Kantarjian et al., 2021). In the pediatric ambit, some of them are under investigation in early-phase trials, particularly in the relapse AML setting (Elgarten and Aplenc, 2020). One of the most promising and studied agents is venetoclax, a B-cell lymphoma-2 (BCL-2) inhibitor that can restore the activation of caspase-dependent apoptosis in malignancies, including AML. While lymphoid malignancies nearly universally overexpress BCL-2 (Stilgenbauer et al., 2016; Ashkenazi et al., 2017), in myeloid leukemias, BCL-2 expression is heterogeneous and not always upregulated, with relapses showing higher percentages of positive expression than those seen at leukemia onset, suggesting that BCL-2-expressing blasts might be those escaping apoptosis in first-line treatments (Bensi et al., 1995; Testa and Riccioni, 2007; Kuusanmäki et al., 2020).

Nevertheless, venetoclax showed limited activity as a single agent in high-risk AML (Konopleva et al., 2016), encouraging the investigation of rationally designed combinations to increase its activity in these subgroups that emerged with the recent FDA approval for venetoclax in combination with hypomethylating agents or low-dose cytarabine for the treatment of newly diagnosed AML in adults who are 75 years of age or older (DiNardo et al., 2019; Wei et al., 2019; DiNardo et al., 2020; Wei et al., 2020a) and with several studies of venetoclax combinations (Bogenberger et al., 2017; Karjalainen et al., 2017; Ma et al., 2019; Pollyea et al., 2019; Fischer et al., 2020; Han et al., 2020).

In this study, we pursued the identification of newly targeted opportunities for KMT2A-rearranged AML that still represent a challenge in the oncohematology field by selecting novel drug combinations. To accomplish this goal, we evaluated the proteomic profile of KMT2A-r AML pediatric patients, unveiling the hyper-activation of BCL-2 antiapoptotic pathway. We re-analyzed the data of high-throughput drug screening previously performed on AML cell lines, identifying AML-KMT2A-r selective drugs. Thus, to tackle KMT2A-r AML, we tested combinations of the novel KMT2A-r identified drugs with the BCL-2 inhibitor venetoclax. Synergistic combinations have been validated in KMT2A-r AML cell lines and *ex vivo* primary KMT2A-r AML samples in a three-dimensional (3D) scaffold mimicking the bone marrow niche, which strengthen the drug efficacy prediction. Our results documented that I-BET151, sunitinib, and thioridazine act synergistically with venetoclax, all converging in different but mitochondrial apoptotic network activation, enhancing leukemia cell death.

## Materials and Methods

### Reverse-Phase Protein Arrays

Reverse-phase protein array (RPPA) analysis was performed as previously described (Sandoval et al., 2012; Aveic et al., 2015).

### *In Vitro* Cell Culture and Treatments

The cell lines NOMO-1, THP-1, and HL-60 (DMSZ, Braunschweig, DE) were maintained in RPMI 1640 (Thermo Fisher Scientific, Waltham, MA, United States) and SHI-1 (DMSZ) in Dulbecco’s modified Eagle’s medium (Thermo Fisher Scientific). All the media were supplemented with 10% fetal bovine serum (FBS; Thermo Fisher Scientific), 2 mM glutamine (Gibco, Life Technologies, CA, United States), and 100 U/ml streptomycin/penicillin (Gibco, Life Technologies).

*Ex vivo* cells were obtained from the bone marrow of pediatric patients affected by *de novo* AML with KMT2A rearrangements, provided by the pediatric OncoHematology Lab of Padova Hospital. Primary cells were cultured in RPMI Medium 1640 with 10% FBS, 2 mM glutamine, and 100 U/ml streptomycin/penicillin, supplemented with cytokines (50 ng/ml hTPO, 50 ng/ml hSCF, 50 ng/ml hFlt3L, 20 ng/ml hIL-3, and 20 ng/ml hIL-6; Miltenyi Biotec, Bergisch Gladbach, Germany).

Venetoclax, I-BET 151, sunitinib, quinacrine, thioridazine, and PD98059 were purchased from Merck Millipore. The KMT2A-MLLT3-rearranged NOMO-1 and THP-1, KMT2A-AFDN-rearranged SHI-1, and non-KMT2A-rearranged HL-60 cell lines were treated at a density of 0.5 × 10^6^/ml at concentrations selected based on the dose–response curve of each drug. In particular, in synergy evaluation, we tested multiple doses as follows: for venetoclax, we tested three doses, including concentrations between IC40 and IC60; for the KMT2A-r AML cell lines, we used 0.2, 1, and 5 μM; and for HL-60, we used 1, 10, and 100 nM. For KMT2A-specific drugs (I-BET151, sunitinib, and quinacrine), we included concentrations ranging from IC25 to IC50 since these drugs would be used in combination for the evaluation of their synergistic abilities. For KMT2A-AFDN-specific drugs, thioridazine and PD98059, we selected the concentration based on our previously published data (Manara et al., 2014a; Tregnago et al., 2020). In the combination experiment, we use one dose of each selected drug: we used the lower dose that resulted as synergistic as shown by the red area of the 2-dimensional contour plot, where we highlighted the selected dose with a yellow star. In HL-60, since the synergy score never showed synergy, we used I-BET151, sunitinib, and quinacrine at the same dose previously selected for the KMT2A-r AML cell lines. The combination index was calculated as reported by Slinker BK *et al*. (CI = EA + EB/EAB, where EAB represents the observed combination effect, E is the effect, A refers to drug A, and B refers to drug B) (Slinker, 1998).

### Cell Viability Assay

Cell viability was evaluated using CellTiter-Glo^®^ assay (Promega Fitchburg, WI) following the guidelines of the manufacturer. Briefly, at the experimental endpoint, 100 μl of treated cells was transferred into the wells of white, flat-bottomed, opaque 96-well plates (Corning Life Sciences, NY, United States), then 100 μl of CellTiter-Glo^®^ reagent was added, and the plates were shaken for 2 min and incubated for 20 min at room temperature. Luminescence was recorded using the Spark^®^ multimode microplate reader (TECAN, Männedorf, Switzerland), with an integration time of 0.1 s per well. Cell viability was evaluated up to 72 h after treatment in dose–response curves, 48 h after treatment in drug combination experiments on AML cell lines, and 24 h after treatment in drug combination experiments on *ex vivo* primary AML cells.

### High-Throughput Screening

High-throughput screening (HTS) was performed as previously described (Tregnago et al., 2020). Drugs specific for KMT2A-rearranged AML were evaluated as those reducing cell viability ≥60% selectively in (6; 11) ML2, SHI-1, and t (9; 11) NOMO-1 and THP-1, but not in HL60, at 10-µM concentration.

### Quantitative Real-Time PCR

Total RNA was isolated using Trizol (Invitrogen—Thermo Fisher Scientific). Then, 1 μg of RNA was reverse-transcribed into cDNA using the SuperScript II system (Invitrogen—Thermo Fisher Scientific) according to the instructions of the manufacturer. The expression of BCL2 mRNA was measured by real-time PCR (RQ-PCR) on an ABI 7900HD platform (Applied Biosystems, Foster City, CA) using the Platinum™ SYBR™ Green qPCR SuperMix (Invitrogen—Thermo Fisher Scientific) and normalized on GUS housekeeping gene using the 2^-ΔΔCt method. The primers are as follows: BCL2 F: GGC​CGT​ACA​GTT​CCA​CAA​A; BCL2 R: AGT​ACC​TGA​ACC​GGC​ACC​T; GUS F: GAA​AAT​ATG​TGG​TTG​GAG​AGC​TCA​TT; GUS R: CCG​AGT​GAA​GAT​CCC​CTT​TTT​A.

### Western Blot

For Western blot analysis, whole cells were lysed in RIPA buffer (Tris-HCl: 50 mM, pH 8, NaCl: 150 mM, Nonidet-P40 1%, sodium deoxycolate: 0.5%, and SDS: 0.1%) and processed for protein expression by Western blot. The following primary antibodies were used: BCL-2 (D55G8, Cell Signaling Technology, Danvers, MA, United States), MCL-1 (Cell Signaling Technology), and GAPDH (GeneTex, Irvine, CA, United States). The horseradish peroxidase–conjugated secondary antibody was either anti-rabbit or mouse (Perkin Elmer, Waltham, MA, United States), and signal was quantified using ImageJ software.

### Mitochondrial Membrane Potential

Mitochondrial membrane potential was measured by using TMRE Assay Kit (ab113852, Abcam, Cambridge, United Kingdom), following the instructions of the manufacturer. Briefly, TMRE (200 nM) diluted in BSA (0.2%)-PBS 1X was added for 20 min at 37°C. The cells were analyzed by flow cytometry using FC500 (Beckman Coulter, Brea, CA).

### Colony-Forming Unit Assay

At 24 h after treatment, 2 × 10^3^ *ex vivo* AML cells were seeded into 500 μl of MethoCult™ (H4534, Stemcell Technologies, Vancouver, Canada) in 24-well plates and incubated at 37°C. After 2–4 weeks of culture, for colony counting, an adequate volume of a 1:6 solution of 3-(4,5-dimethylthiazol-2-yl)-2,5-diphenyltetrazolium bromide (Sigma-Merck Millipore) in Hanks’ balanced salt solution was added to the semisolid medium, and images were acquired by an optical microscope with camera.

### *In Vitro* 3D-AML Treatments

The AML-MSC isolation and culture, 3D scaffold specifications, and 3D culturing setup were previously described (Borella et al., 2021). Briefly, AML-MSCs were seeded in the scaffold and cultured in StemMACSTM MSC Expansion Media (Miltenyi Biotec) for MSC expansion for 5 days. Then, AML cells were added to the scaffold in proper medium and exposed to the best drug combinations that resulted from the monoculture setting. We used the highest synergistic doses found by ZIP synergy analysis, tested to be safe in MSCs, that are I-BET151 (2 µM), sunitinib (5 µM), and thioridazine (10 µM). Venetoclax was used (1 µM). Treatment was conducted for 48 h.

### 3D Cell Viability Assay

Cell viability was measured using CellTiter-Glo^®^ 3D reagent (Promega) according to the instructions of the manufacturer at 48 h after treatment. Briefly, the scaffolds were individually transferred into wells of white, flat-bottomed, opaque 96-well plates (Corning Life Sciences) with 100 μl of RPMI; then, 100 μl of CellTiter-Glo^®^ 3D reagent was added into each well. The plates were shaken for 5 min to induce scaffold and cell lysis. The samples were then incubated for an additional 20 min in the dark, at room temperature, to stabilize the bioluminescent signal, which was then recorded using Spark^®^ multimode microplate reader (TECAN), with an integration time of 0.1 s per well. Cell viability was compared to the control sample [scaffold with the same cells but treated with dimethyl sulfoxide (DMSO)].

In co-treatment experiments, the combination index was calculated as reported by Slinker BK *et al*. (CI = EA + EB / EAB, where EAB represents the observed combination effect, E is the effect, A refers to drug A, and B refers to drug B) (Slinker, 1998).

### Data Evaluation and Statistical Analyses

The *t*-test was adopted for significance between differences in means when two groups were evaluated, after a preliminary testing of normal distribution of data. ANOVA test was performed when comparing more than two groups, applying Bonferroni correction for multiple statistical hypotheses testing. Graphs and associated statistical analyses were generated using GraphPad Prism 8 (GraphPad, La Jolla, CA). All data are presented as mean ± standard error of the mean (SEM), with *p-value <0.05, **p-value <0.01, ***p-value <0.001, and ****p-value <0.0001 considered statistically significant.

## Results

### BCL-2 Family Antiapoptotic Proteins in Pediatric AML

**FIGURE 1:**
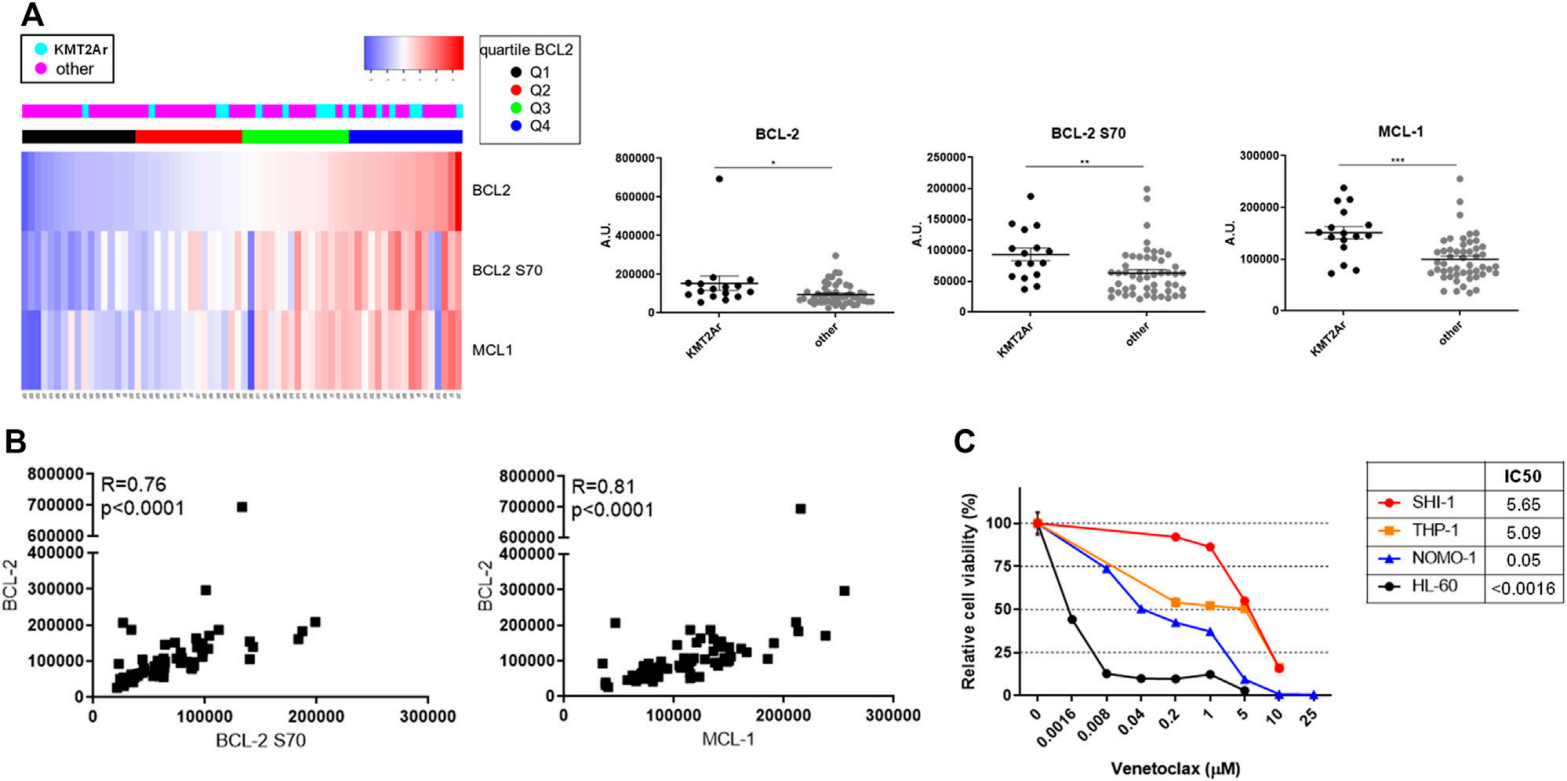
Expression of antiapoptotic proteins of BCL-2 family in pediatric acute myeloid leukemia (AML). (**A**) Supervised analysis according to BCL-2 expression (left panel) and dot plots (right panel) showing BCL-2, BCL-2 S70, and MCL-1 protein expression of a cohort of 66 AML pediatric patients subcategorized in KMT2A-rearranged AML and non-KMT2A-rearranged AML, analyzed with the reverse-phase protein array method. Quartiles refer to BCL-2 expression. Dot plots show the mean ± SEM. A.U., arbitrary units. (**B**) Pearson correlation between BCL-2 S70 (*X*-axis) and BCL-2 (*Y*-axis) in the upper panel and MCL-1 (*X*-axis) and BCL-2 (*Y*-axis) in the lower panel; *p* < 0.00001. (**C**) Dose–response curve of growing concentrations of venetoclax in KMT2A-rearranged AML (SHI-1, THP-1, and NOMO-1) and non-KMT2A-rearranged AML (HL-60) cell lines at 72 h after treatment (*n* = 2).

**FIGURE 2:**
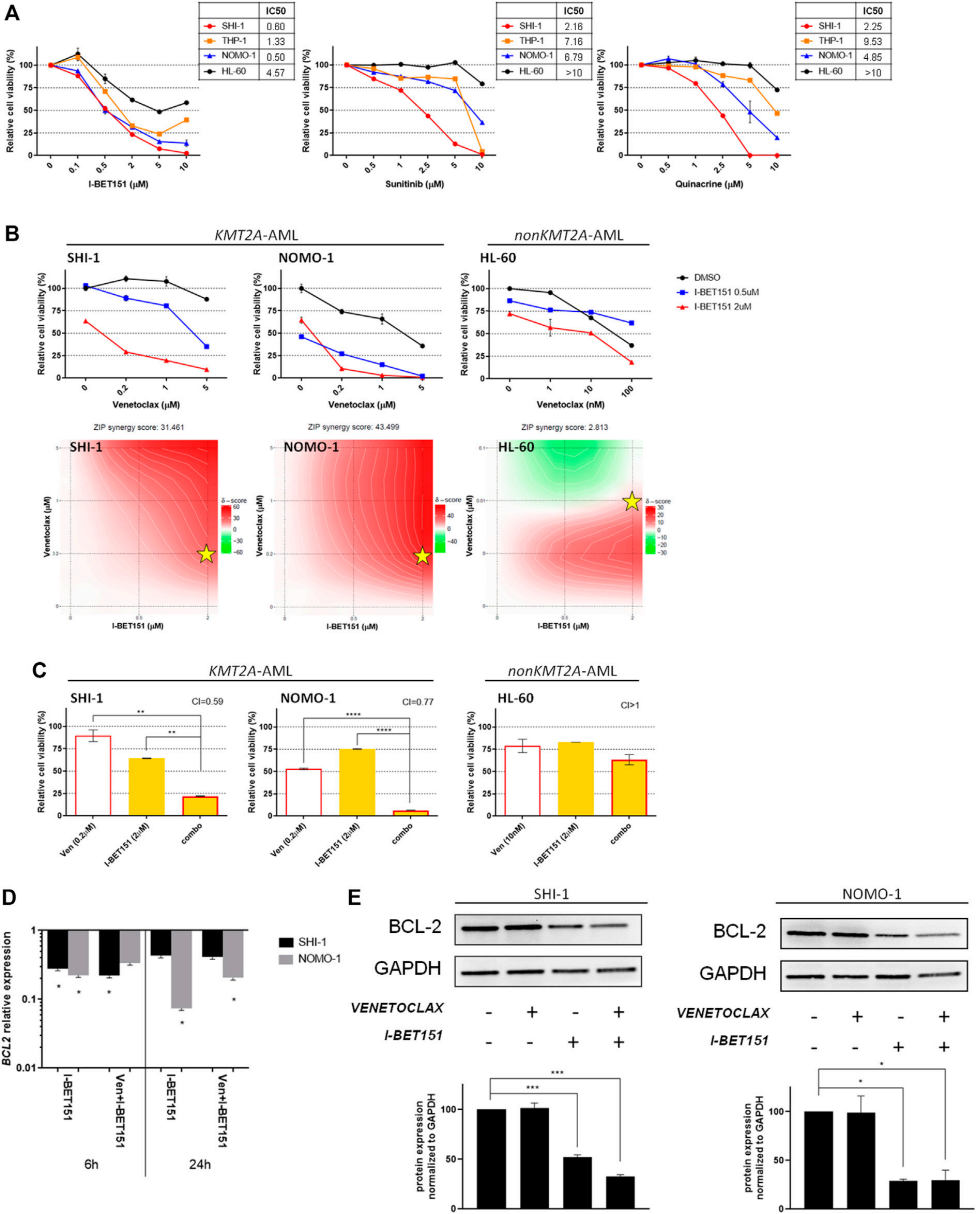
Combination of venetoclax and I-BET151. (**A**) Dose–response curve of growing concentrations of I-BET151, sunitinib, and quinacrine in KMT2A-rearranged acute myeloid leukemia (AML; SHI-1, THP-1, and NOMO-1) and non-KMT2A-rearranged AML (HL-60) cell lines at 72 h after treatment (*n* = 2). (**B**) Cell viability of SHI-1 and NOMO-1 (KMT2A-rearranged) or HL-60 (non-KMT2A-rearranged) after treatment with I-BET151 combined with venetoclax at 48 h after treatment. The synergy scores were represented by pseudocoloring 2-dimensional contour plots over the dose matrix (red indicates synergy and green indicates antagonism) and calculated using the ZIP model (synergy when >10, *n* = 2). Stars indicate the concentrations selected for subsequent experiments. (**C**) Cell viability of SHI-1 and NOMO-1 (KMT2A-rearranged) or HL-60 (non-KMT2A-rearranged) after treatment with venetoclax, I-BET151, or the combination at 48 h after treatment (CI, combination index; synergy when CI <1. ANOVA test was performed by applying Bonferroni correction for multiple statistical hypotheses testing. **p < 0.01, ****p < 0.0001; *n* = 2). (**D**) *BCL2* expression measured by RQ-PCR at 6 and 24 h post-treatment in SHI-1 and NOMO-1 with respect to control. ANOVA test was performed by applying Bonferroni correction for multiple statistical hypotheses testing. *p < 0.05; *n* = 2 (**E**) BCL-2 levels measured at 48 h post-treatment in SHI-1 and NOMO-1. Histograms report the quantification normalized to GAPDH. ANOVA test was performed by applying Bonferroni correction for multiple statistical hypotheses testing. *p < 0.05, ***p < 0.001; *n* = 2.

We analyzed the BCL-2, BCL-2 S70, and MCL-1 protein levels in a cohort of 66 pediatric AML by RPPA analysis (Aveic et al., 2015), and we subdivided the patients by BCL-2 protein expression quartiles. We found that KMT2A-rearranged AML patients were prevalent in the higher quartiles Q3 + Q4 with respect to the lower Q1 and Q2 (KMT2A-r cases: Q3 + Q4, *n* = 12, 75% *vs*. Q1 + Q2, *n* = 4, 25%; Figure 1A, Supplementary Table S1). Overall, the KMT2A-rearranged AML patients have significantly high levels of BCL-2, phospho-BCL-2 S70, and MCL-1 (Figure 2A; **p < 0.01, ***p < 0.001). Of note is the fact that we found a significant correlation between BCL-2 and BCL-2S70 (*R* = 0.76, *p* < 0.00001) and between BCL-2 and MCL-1 (*R* = 0.81, *p* < 0.00001), describing that there was an active anti-apoptotic pathway in KMT2A-AML (Figure 1B). Conversely, patients with isolated core binding factor rearrangements that are well responders to chemotherapy were prevalent in the Q1 + Q2 quartiles (Q1 + Q2, *n* = 15, 65% *vs*. Q3 + Q4, *n* = 8, 35%).

We tested the BCL-2 inhibitor venetoclax in a panel of three KMT2A-r AML cell lines, namely, t (6; 11) SHI-1 and t (9; 11) NOMO-1 and THP1 and in a non-KMT2A cell line, HL60. We found that the SHI-1 and THP1 cell lines were resistant (IC50 >5 µM) to the treatment compared to the HL60 that was sensitive (IC50 <0.0016 µM), with NOMO1 cells responding at intermediate levels (IC50 = 0.05 µM; Figure 1C, Supplementary Table S2), supporting the KMT2A-rearranged cells as suitable for the anti-apoptotic pathway to be tackled.

### Selection of KMT2A-AML-Specific Drugs and Combination Strategy

We considered to increase the susceptibility of KMT2A-AML to venetoclax by combining it with other KMT2A-specific agents. To identify the most suitable drugs, we re-analyzed the data of the high-throughput chemical screening previously performed (Tregnago et al., 2020), filtering 1,280 drugs for being effective in reducing cell proliferation to ≤60% in KMT2A-AF6- and KMT2A-AF9-rearranged AML cell lines, but not in HL60 (Supplementary Figure S1). This strategy uncovered three candidate drugs for KMT2A-AML treatment improvement, including I-BET 151, a BET bromodomain inhibitor which inhibits BRD4, BRD2, and BRD3 as previously identified for KMT2A-r AML (Fu et al., 2015), sunitinib, a multitargeted kinase inhibitor (Papaetis and Syrigos, 2009), and quinacrine, an antimalarial drug that inhibits NFκB suppression of p53 (Oien et al., 2021), both being never explored in the KMT2A-r AML context. The dose–response curve confirmed the HTS results, showing a higher sensitivity of the KMT2A-AML cells to all the three drugs when compared to the HL60 (Figure 2A).

To assess the pharmacological interactions between venetoclax and KMT2A-specific drugs, incremental doses were applied based on the IC50 value of each drug (Supplementary Table S3) on SHI-1 and NOMO1 as representatives of KMT2A-AML and on HL60 as non-KMT2A-AML. Drug synergy was interrogated by using SynergyFinder application (Ianevski et al., 2021) (synergy score <−10: antagonistic interaction; >−10 and <10: additive interaction; >10: synergistic interaction). The results showed a strong synergism between venetoclax and I-BET 151 in reducing KMT2A-AML cell proliferation (synergy score: 31.461 in SHI-1, 43.499 in NOMO1, and 2.812 in HL60; Figure 2B), confirmed by a significant combination index (CI) in all combination treatments (CI = 0.59 in SHI-1, 0.77 in NOMO1, and >1 in HL60; Figure 2C; **p < 0.01, ****p < 0.0001). Thus, we explored BCL-2 RNA and protein expression after I-BET 151 treatment, as BCL-2 is a direct KMT2A target whose transcription is dependent on BET family protein placement on chromatin (Dawson et al., 2011), and we found that during treatment, BCL-2 was significantly decreased (Figures 2D,E, *p < 0.05, ***p < 0.001) in both RNA and protein expression, sensitizing KMT2A-AML to venetoclax treatment.

**FIGURE 3:**
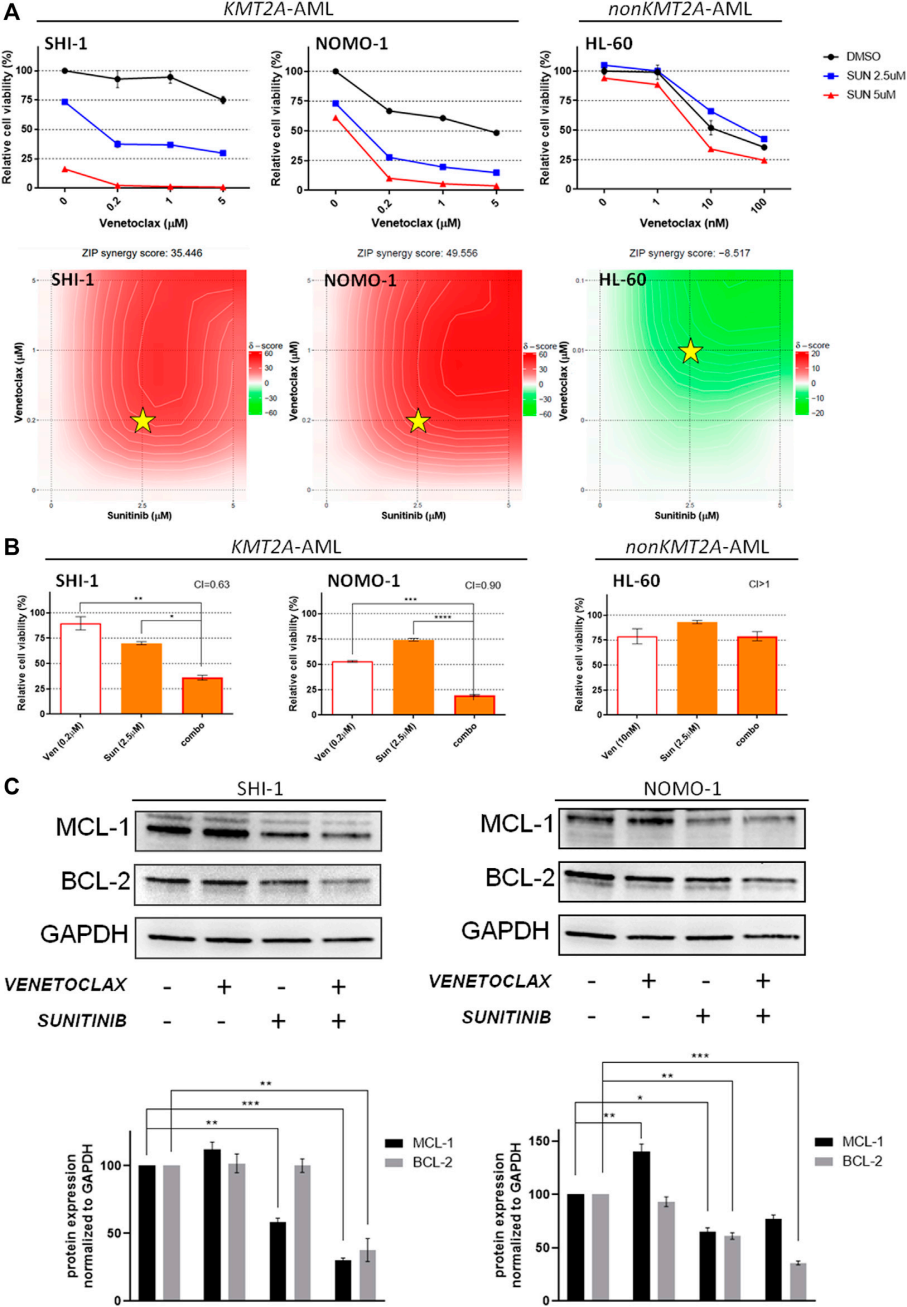
Combination of venetoclax and sunitinib. (**A**) Cell viability of SHI-1 and NOMO-1 [both KMT2A-rearranged acute myeloid leukemia (AML)] and HL-60 (non-KMT2A-rearranged AML) after treatment with sunitinib combined with venetoclax at 48 h after treatment. The synergy scores were represented by pseudocoloring 2-dimensional contour plots over the dose matrix (red indicates synergy and green indicates antagonism) and calculated using the ZIP model (synergy when >10, *n* = 2). Stars indicate the concentrations selected for subsequent experiments. (**B**) Cell viability of SHI-1 and NOMO-1 (KMT2A-arranged) or HL-60 (non-KMT2A-rearranged) after treatment with venetoclax, sunitinib, or the combination at 48 h after treatment (CI, combination index; synergy when CI <1. ANOVA test was performed by applying Bonferroni correction for multiple statistical hypotheses testing. *p < 0.05; **p < 0.01, ***p < 0.001; ****p < 0.0001; *n* = 2). (**C**) MCL-1 and BCL-2 levels measured at 48 h post-treatment in SHI-1 and NOMO-1. Histograms report the quantification normalized to GAPDH. ANOVA test was performed by applying Bonferroni correction for multiple statistical hypotheses testing. *p < 0.05, **p < 0.01, ***p < 0.001; *n* = 2.

We tested the venetoclax and sunitinib combination with the same approach, finding a strong synergism in KMT2A-AML (Figure 3A; synergy score: 35.446 in SHI-1, 49.556 in NOMO1, and - 8.517 in HL60), and the combination treatment resulted in significantly decreasing the cell viability (CI = 0.63 in SHI-1, 0.90 in NOMO1, and >1 in HL60; Figure 3B; *p < 0.05, **p < 0.01, ***p < 0.001, ****p < 0.0001). In this case, a more in-depth molecular analysis of the mechanisms underlying the cellular response showed that sunitinib decreased the MCL-1 expression more than the BCL-2 (Figure 3C; *p < 0.05, **p < 0.01, ***p < 0.001), supporting that KMT2A-AML cells were susceptible to venetoclax treatment due to the effects induced in MCL-1.

**FIGURE 4:**
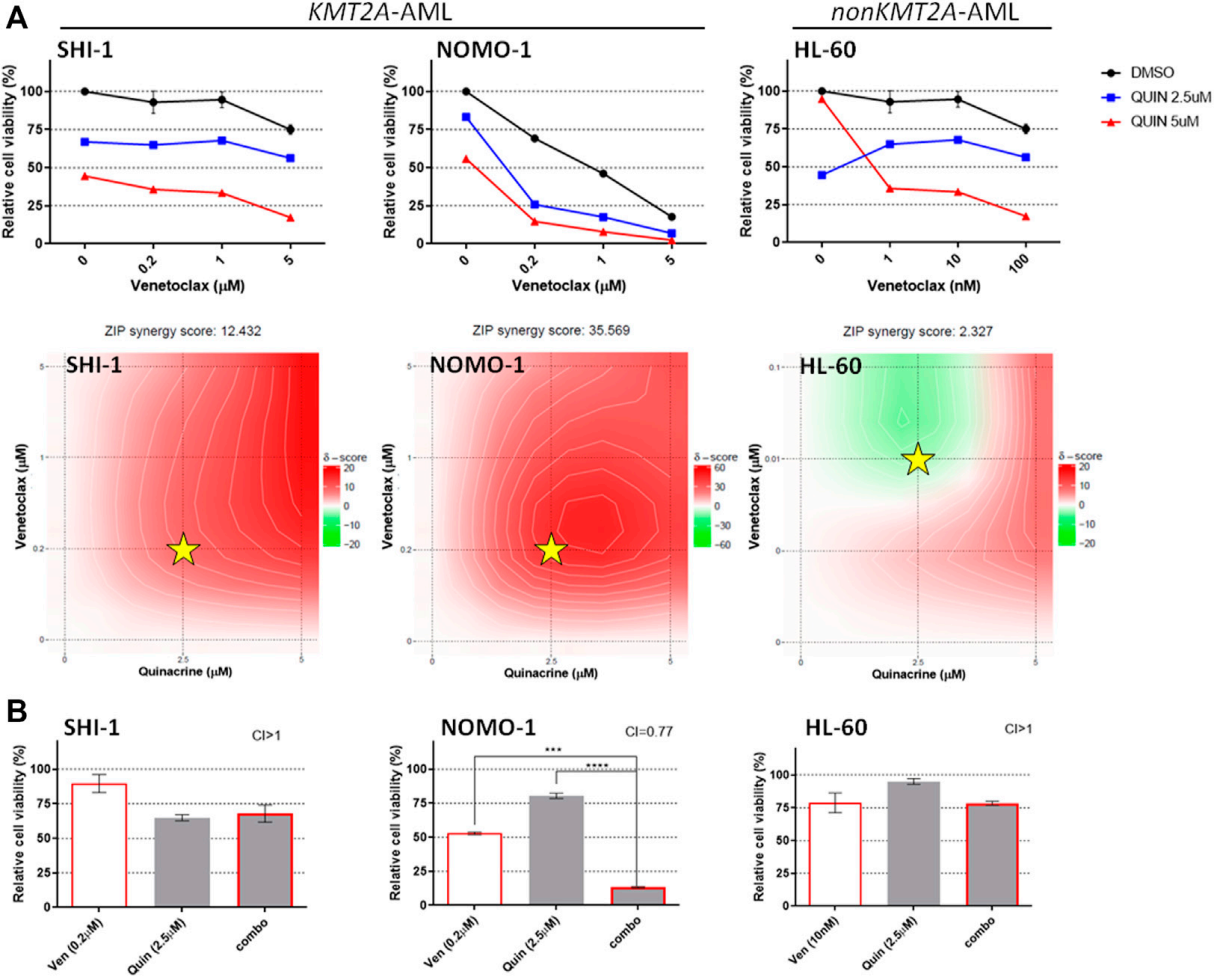
Combination of venetoclax and quinacrine. (**A**) Cell viability of SHI-1 and NOMO-1 [both KMT2A-rearranged acute myeloid leukemia (AML)] and HL-60 (non-KMT2A-rearranged AML) after treatment with quinacrine combined with venetoclax at 48 h after treatment. The synergy scores were represented by pseudocoloring 2-dimensional contour plots over the dose matrix (red indicates synergy and green indicates antagonism) and calculated using the ZIP model (synergy when >10, *n* = 2). Stars indicate the concentrations selected for subsequent experiments. (**B**) Cell viability of SHI-1 and NOMO-1 (KMT2A-arranged) or HL-60 (non-KMT2A-rearranged) after treatment with venetoclax, quinacrine, or the combination at 48 h after treatment (CI, combination index; synergy when CI <1. ANOVA test was performed by applying Bonferroni correction for multiple statistical hypotheses testing. ***p < 0.001, ****p < 0.0001; *n* = 2).

Conversely, we found synergism between venetoclax and quinacrine only in NOMO-1 (Wei et al., 2020b), whereas in SHI-1 the score was just barely synergistic (Figure 4A; synergy score: 12.432 in SHI-1, 35.569 in NOMO1, and 2.327 in HL60). Since quinacrine-induced cell death and mitochondrial depolarization were described to be mediated by MAPK-elicited BCL2 downregulation and suppressed by constitutively active MEK-1 over-expression (Changchien et al., 2015), we hypothesize that RAS pathway overactivation in t (6; 11)-r AML (Manara et al., 2014a) might prevent quinacrine-induced BCL-2 downregulation, thus avoiding synergy.

Consistently, the combination treatment resulted to be synergistic only in NOMO-1 (Figure 4B; CI >1 in SHI-1, 0.77 in NOMO1, and >1 in HL60; ***p < 0.001, ****p < 0.0001), excluding this combination of drugs from further analysis.

### Combination Strategy in KMT2A-AF6-Rearranged AML

**FIGURE 5:**
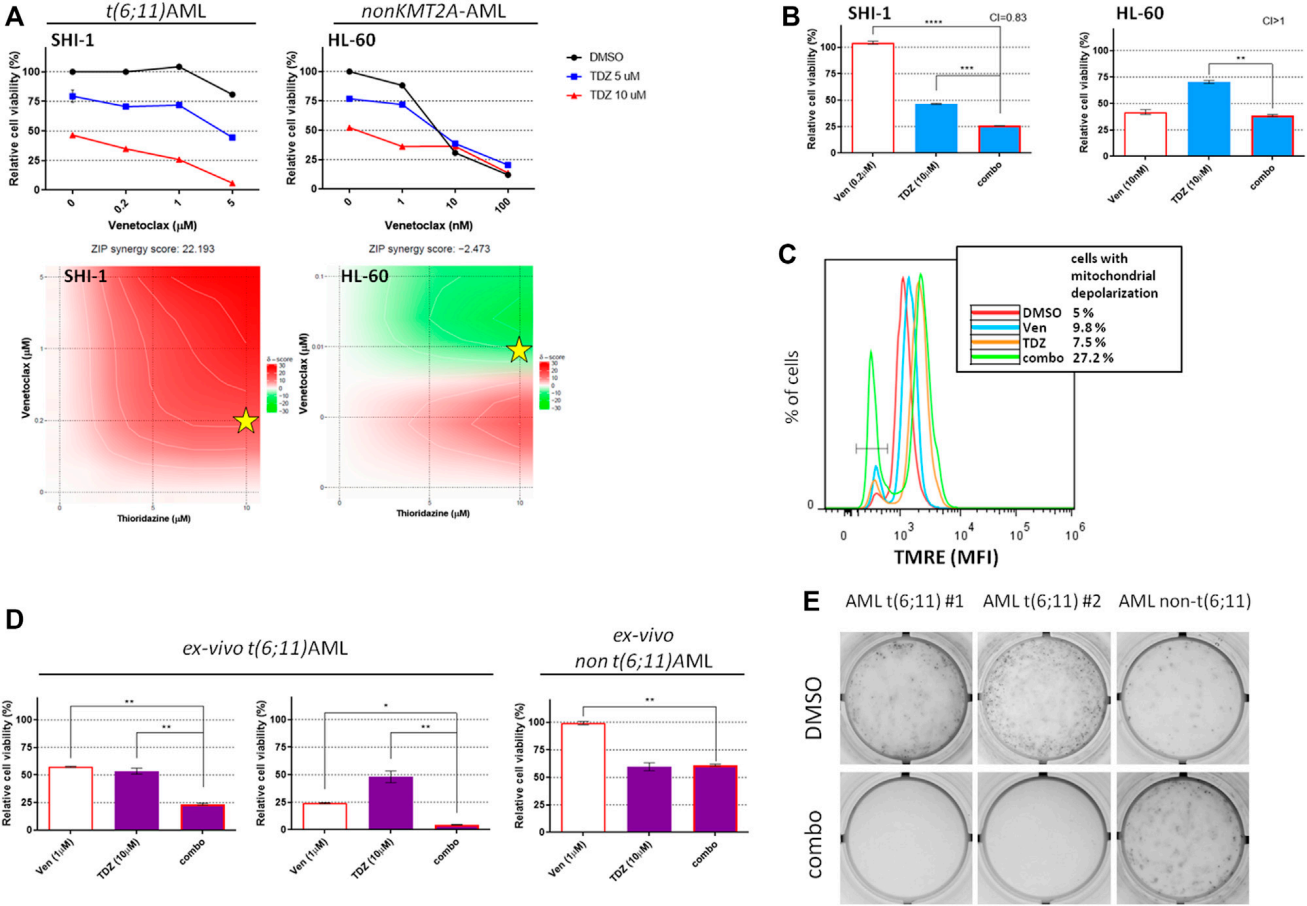
Combination of venetoclax and thioridazine in t(6;11)-rearranged acute myeloid leukemia (AML). (**A**) Cell viability of t (6; 11) SHI-1 and non-t (6; 11) HL-60 after treatment with thioridazine (TDZ) combined with venetoclax (Ven) at 48 h after treatment. The synergy scores were represented by pseudocoloring 2-dimensional contour plots over the dose matrix (red indicates synergy and green indicates antagonism) and calculated using the ZIP model (synergy when >10, *n* = 2). Stars indicate the concentrations selected for subsequent experiments. (**B**) Cell viability of t (6; 11) SHI-1 and non-t (6; 11) HL-60 after treatment with venetoclax, thioridazine, or the combination at 48 h after treatment (CI, combination index; synergy when CI <1. ANOVA test was performed by applying Bonferroni correction for multiple statistical hypotheses testing. **p < 0.01, ***p < 0.001; ****p < 0.0001; *n* = 2). (**C**) Mitochondrial depolarization evaluated by tetramethylrhodamine ethyl fluorescence measurement at 20 h after Ven (5 µM), TDZ (10 µM), or combination treatment compared with dimethyl sulfoxide in SHI-1 (*n* = 2). (**D**) Cell viability of PDX-derived *ex vivo* t (6; 11) and non-t (6; 11) AML after treatment with venetoclax (1 µM), thioridazine (10 µM) or the combination at 24 h after treatment. ANOVA test was performed by applying Bonferroni correction for multiple statistical hypotheses testing.*p < 0.05; **p < 0.01. (**E**) Colony-forming assay performed on viable *ex vivo* cells seeded at 24 h after the combination treatment [venetoclax (1 µM) + thioridazine (10 μM), *n* = 2].

Since the t (6; 11)-r AML is known to be a peculiar KMT2A-r subgroup (Balgobind et al., 2009; Pigazzi et al., 2011; Pession et al., 2013), we previously identified that thioridazine and PD98059 selectively reduced KMT2A-AF6 AML cell proliferation and described their mechanism of action (Manara et al., 2014a; Tregnago et al., 2020). Here we tested their activity in combination with venetoclax. The results showed that the combination of venetoclax with thioridazine is strongly synergic (Figure 5A; synergy score: 22.193 in SHI-1 and -2.473 in HL60), and the combination treatment resulted in significantly decreasing the cell viability (CI = 0.83 and >1 in SHI-1 and HL60, respectively; Figure 5B; **p < 0.01, ***p < 0.001, ****p < 0.0001). We previously demonstrated that apoptosis was driven by Ca^2+^ influx-induced mitochondrial depolarization that occurred after thioridazine treatment. Therefore, we looked at the mitochondrial potential status, finding that the combined treatment led to a more rapid mitochondrial depolarization, triggering greater cytotoxicity (cells with depolarized mitochondria: 5% with DMSO, 9.8% with venetoclax, 7.5% with thioridazine, and 27.2% with combination; Figure 5C). Finally, we would consider these novel combination strategies in *ex vivo* AML cells derived from *de novo* AML patient-derived xenografts (PDXs). A dose–combination curve confirm that the t (6; 11)-AML samples were more sensitive to thioridazine treatment compared with the non-t (6; 11) ones (Supplementary Figure S2, Table S4), and the combination with venetoclax further and selectively reduced cell viability in this genetic subgroup (Figure 5D; *p < 0.05, **p < 0.01). Notably, we revealed that the combination treatment of venetoclax + thioridazine completely abrogated the cell clonogenic capacity of t (6; 11)-rearranged AML cells, supporting this strategy to be useful for a complete blast clearance (Figure 5E).

On the contrary, although the combination of PD98059 + venetoclax was more effective in the t (6; 11) cell line, it did not result to be synergistic (Supplementary Figure S3; synergy score: 2.479 in SHI-1 and -3.531 in HL60), and for this reason, we excluded this combination of drugs from further analysis.

### Best Combination Strategy in KMT2A-AML in a 3D System

**FIGURE 6:**
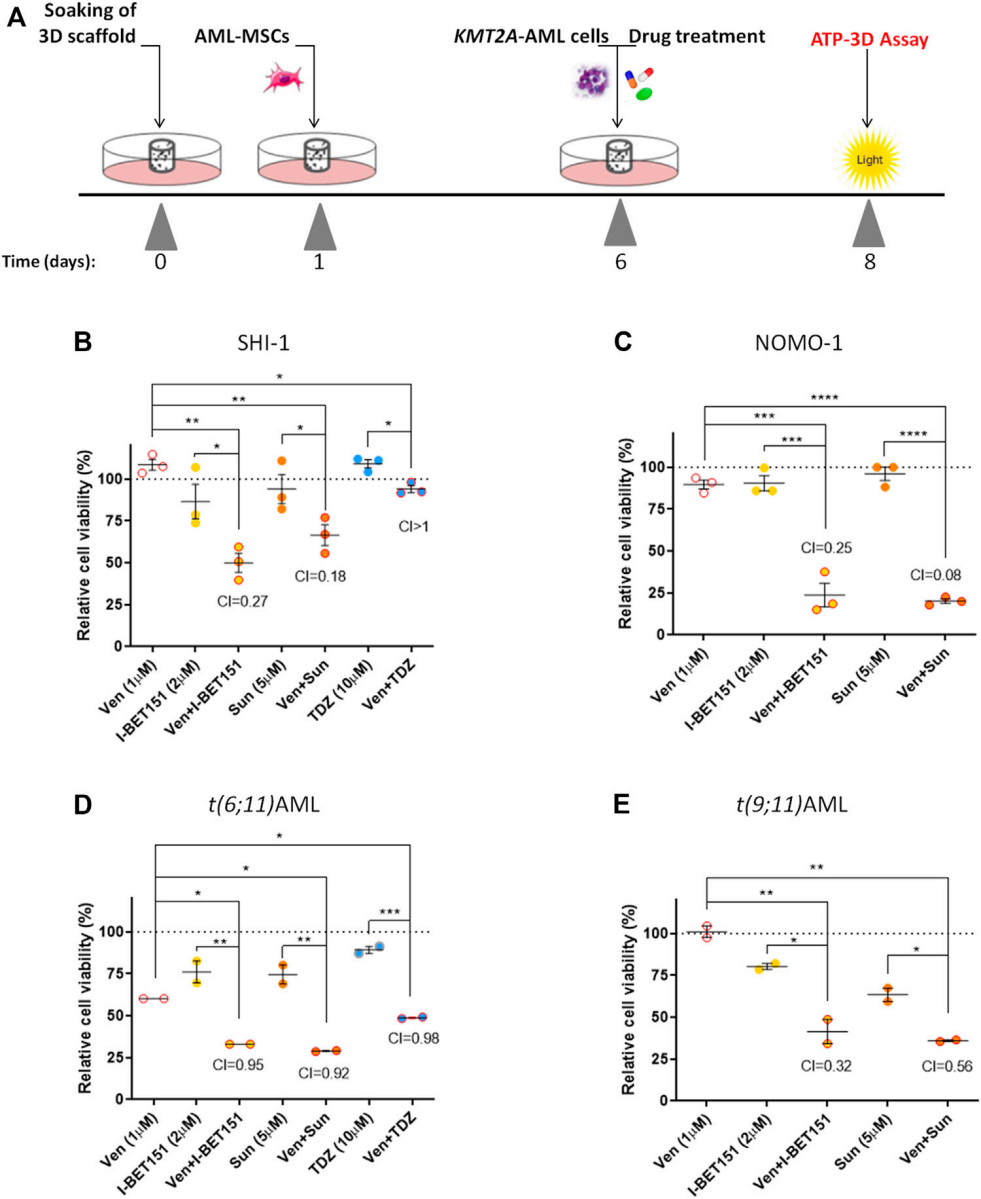
Combination treatments in 3D model. (**A**) Scheme of the *in vitro* 3D culture setup procedure: hydroxyapatite/collagen scaffolds were allowed to soak for 24 h, then seeded with acute myeloid leukemia (AML)-MSCs (*t* = 1), and cultured for a further 5 days prior to adding of KMT2A-rearranged AML cells and performing the drug treatment (*t* = 6). Cell viability was evaluated in the 3D system at 48 h after treatment (*t* = 8) by ATP 3D assay. (**B**–**D**) Cell viability of 3D system analyzed at 48 h after drug treatment with venetoclax (1 μM), I-BET151 (2 μM), sunitinib (5 µM), and thioridazine (10 μM) normalized to the respective controls (dimethyl sulfoxide; *n* = 3) in SHI-1 and NOMO-1 cell lines (**B**, **C**) and AML primary samples (**D**, **E**). CI, combination index; synergy when CI <1. ANOVA test was performed by applying Bonferroni correction for multiple statistical hypotheses testing. *p < 0.05; **p < 0.01; ***p < 0.001; ****p < 0.0001.

To validate our results, we took advantage of an innovative three-dimensional (3D) culture model that we recently developed; it consists of a 3D biomimetic scaffold made of hydroxyapatite/collagen (70/30%) (Tampieri et al., 2008) that allowed long-term AML co-culture with mesenchymal stromal cells (MSCs) derived from the bone marrow (BM) of leukemia patients at diagnosis of *de novo* AML (namely, AML-MSCs), recapitulating the crucial physiological aspects of leukemia niche (Borella et al., 2021). This 3D system was demonstrated to be a *bona fide* model, wherein robust and reliable drug screening is performed due to the recapitulation of the BM microenvironment. Therefore, we tested the drugs selected above in SHI-1 and NOMO-1 cells seeded in the 3D system together with AML-MSCs, following a sequential cell seeding schema of long-term AML cultures in 3D (Figure 6A). Of note is that since we previously documented a different drug active concentration in 2D or 3D models, with a generally higher dose needed to induce the same cell death in the 3D model, here we increased the doses of the compounds, verifying that they did not affect the viability of AML-MSCs in co-cultures, with MSCs always proliferating >70% with drugs used alone or in combination (Supplementary Figure S4). Blasts co-cultured in 3D and treated for 2 days showed that either venetoclax + I-BET151 or venetoclax + sunitinib was able to significantly reduce the cell viability of both SHI-1 and NOMO-1 (Figures 6B,C; SHI: CI = 0.27 with Ven + IBET151, CI = 0.18 with venetoclax + sunitinib; NOMO-1: CI = 0.25 with venetoclax + IBET151; CI = 0.08 with venetoclax + sunitinib; *p < 0.05, **p < 0.01, ***p < 0.001, ****p < 0.0001), confirming the strong synergy of these drug combinations. Finally, we performed treatments in the 3D system seeded with *ex vivo* primary AML samples and demonstrated a greater effect in reducing leukemia cell proliferation when drugs are used in combination on t (6; 11) and t (9; 11) KMT2A-r AML (Figure 6D; t (6; 11)AML: CI = 0.95 with Ven + IBET151, CI = 0.92 with venetoclax + sunitinib, CI = 0.98 with venetoclax + thioridazine; t (9; 11)AML: CI = 0.32 with venetoclax + IBET151; CI = 0.56 with venetoclax + sunitinib; *p < 0.05, **p < 0.01, ***p < 0.001; Supplementary Figure S5). All the combinations tested are summarized in Table 1.

**TABLE 1:** List of the combinations tested, the molecular mechanisms identified for the observed synergy, and the setting where the treatments were performed.

Molecular rearrangement	Combination tested	Synergy	Molecular mechanism identified	*In vitro* drug treatment		
				AML cell lines (SHI-1, NOMO-1, HL-60)	3D model	
					AML cell lines (SHI-1, NOMO-1)	*Ex vivo* primary AML cells
KMT2A- rearranged AML	Venetoclax + I-BET151	Yes	Decreased BCL-2	X	X	X
	Venetoclax + sunitinib	Yes	Decreased MCL-1 and BCL-2	X	X	X
	Venetoclax + quinacrine	No	-	X	-	-
KMT2A-AFDN- rearranged AML	Venetoclax + thioridazine	Yes	Mitochondria depolarization	X	X	X
	Venetoclax + PD98059	No	-	X	-	-

## Discussion

In the field of pediatric AML, the need to improve survival while decreasing relapse occurrence and treatment-related toxicity dictates the great effort that is continually being spent to find novel therapeutic approaches. To reach this goal, it is necessary to consider the high intra- and inter-AML complexity both at the genetic and clinical points of view.

Here we aimed at identifying new therapeutic strategies for a specific genetic AML subgroup, taking into account differences at the molecular and pharmacological levels and regarding cell–cell interaction using a model that closely resembles the leukemic niche microenvironment with stromal component. We would test the possible use of venetoclax in the pediatric AML setting and evaluated the BCL-2 levels in a cohort of pediatric AML samples, finding the majority of KMT2A-rearranged AML patients to be allocated in Q3 + Q4 quartiles and to have significantly high levels of BCL-2, phospho-BCL-2 S70, and MCL-1, suggesting that this antiapoptotic signaling is particularly upregulated in this subgroup. Interestingly, the BCL-2 S70 phosphorylated form and MCL-1 were also found to be overexpressed in the same KMT2A-r AML. It was demonstrated that high levels of phospho-BCL-2 inhibit the effects of venetoclax on the displacement of BAX and BIM from BCL-2, thereby suppressing mitochondrial apoptosis, due to a structural alteration in the BH3-binding groove induced by the phosphorylation of BCL-2 (Ruvolo et al., 2001; Song et al., 2016). Moreover, MCL-1 upregulation may serve as one of the potential mechanisms of cellular resistance to venetoclax (Wei et al., 2020b). Therefore, we investigated KMT2A-rAML cell lines and found that they were resistant or less responsive to venetoclax treatment. However, recent studies have shown that, when combined with either chemotherapy or a panel of targeted drugs, venetoclax resulted in higher response rates with encouraging remission durations in adult AML patients, even when there was a poor response to conventional induction chemotherapy (Wei et al., 2020a; DiNardo et al., 2020). Here we applied a strategy that consists in combining venetoclax with highly selective drugs for KMT2A-r AML. The selective drugs were identified using HTS of chemical compounds partly approved by the FDA or in clinical development, filtered with a very restrictive pipeline that allowed a high refinement to select the drugs specific for the KMT2A rearrangement. The compounds that met our criteria, I-BET151, sunitinib, and quinacrine, rely on different mechanisms of action; however, they all converge in deregulating BCL-2 family proteins, revealing that KMT2A-r AML involved antiapoptotic factors and thus their targeting as a strategy to trigger their death, even more when combined with BCL-2 inhibitor venetoclax. As a result, we documented that I-BET151 and sunitinib, in combination with venetoclax, were selectively synergistic in KMT2A-r cell lines. Notably, a substantial impairment of cell viability was observed even if the concentration of the single drugs was lower than the IC50 value. This is particularly important for venetoclax, for whom a low concentration is recommended to maintain BCL-2 selectivity over BCL-XL to allow BCL-2 antagonism, thus avoiding platelet toxicity (Souers et al., 2013). I-BET151 is a compound that inhibits the bromodomain and extra terminal (BET) protein BRD4 and was previously reported to be particularly efficient on KMT2A-r leukemias (Dawson et al., 2011; Zuber et al., 2011), confirming the robustness of our high-throughput screening. In our cell lines, drug treatment inhibits the transcription of the *BCL2* gene, lowering the BCL-2 protein and significantly sensitizing the cells to venetoclax treatment. Differently, sunitinib is a small molecule with selectivity for PDGFR, VEGFR1, VEGFR2, KIT, and FLT3 (Mendel et al., 2003; O’Farrell et al., 2003), which is currently approved for treating renal cell carcinoma, gastrointestinal stromal tumor, and AML (Wu et al., 2018); however, until now, it has been considered only for FLT3 mutated AML patients in combination with chemotherapy (Fiedler et al., 2015). In this work, we found not only that sunitinib is selective for KMT2A-r cells but that, when combined with venetoclax, it was able to further sensitize cells to treatment, significantly impairing cell viability. We reported that the underlying mechanism is decreased MCL-1 expression, mediating the venetoclax effects. This mechanism was observed also in chronic leukemia cells (Oppermann et al., 2016) and might represent a notable strategy to overcome venetoclax resistance, which often depends on MCL-1 upregulation. Overall, in the KMT2A-rearranged subgroup of pediatric AML, a molecular-based approach may predict the best combination partner of venetoclax. Briefly, we suggest for AML patients with high BCL-2 levels the venetoclax + I-BET151 combination, which decreases BCL2 gene transcription and BCL-2 protein levels, whereas for AML patients with high MCL-1 levels, the venetoclax + sunitinib combination would be considered for its ability to affect MCL-1 levels.

A further refinement of KMT2A treatments is based on the HTS focused on t (6; 11) KMT2A-AFDN-rearranged cases that allowed the identification of thioridazine as the most selective compound inducing the cytoskeletal remodeling of blast cells that led to Ca^2+^ influx, triggering apoptosis through mitochondrial depolarization due to chimera-driven AFADIN delocalization into the nucleus (Tregnago et al., 2020). Given that venetoclax action converges on the mitochondria which actively participate in apoptosis, we monitored the mitochondrial potential after the thioridazine + venetoclax combined treatment, observing an exacerbation of the mitochondrial depolarization that resulted in high synergism of treatment, supporting the combination venetoclax + thioridazine as a useful strategy to be involved in further analyses for AML patients with KMT2A-AFDN fusion with either high BCL-2 or MCL-1 levels. To sustain these findings, we would comply with the emerging need to study AML and its pharmacological targeting in suitable models that take into account the interplay between blasts and the microenvironment (Fang and Eglen, 2017; Langhans, 2018; Cartledge Wolf and Langhans, 2019). For this purpose, we recently developed and characterized a 3D model for AML long-term treatment cultures by seeding primary mesenchymal stromal cells derived from the bone marrow of AML patients (namely, AML-MSCs) into a scaffold that resembles the composition and structure of the bone trabecules of the niche. In this 3D system, we described interactions between leukemic cells and stromal cells which play a critical role in AML proliferation and drug response (Borella et al., 2021). Therefore, we validated novel selected drug combinations in this setting, either with cell lines or primary samples, confirming a significant improvement mediated by combined treatments in reducing leukemia burden.

In summary, the combination of venetoclax with I-BET151, sunitinib, or thioridazine dramatically decreases cell viability in KMT2A-r AML. This anti-leukemia efficacy is associated with the simultaneous inhibition of BCL-2 by venetoclax and the downregulation of anti-apoptotic proteins or disrupting mitochondrial homeostasis, supporting that enhancing the mitochondrial pathway targeting could be a good strategy to sensitize resistant AML to venetoclax. Our 3D system co-cultured with primary MSCs and KMT2A-r blasts confirmed that our combinations are effective and predictable, allowing further rational prioritization of these compounds to be included in preclinical trials.

Overall, this study provides a rationale for evaluating therapy cocktails in 3D models and preclinical studies to accelerate the introduction of new compounds to treat the life-threatening KMT2A-AML subgroup of pediatric AML.

## Data Availability

The original contributions presented in the study are included in the article/Supplementary Material. Further inquiries can be directed to the corresponding author.
