# Supplementary material for: Novel Compounds Synergize With Venetoclax to Target KMT2A-Rearranged Pediatric Acute Myeloid Leukemia
Source: Front Pharmacol. 2022 Jan 27;12:820191. doi: 10.3389/fphar.2021.820191 (PMC8830338; doi:10.3389/fphar.2021.820191)
Supplement: Supplementary file 1 [file DataSheet1.docx]

Supplementary Material

# Supplementary Figures


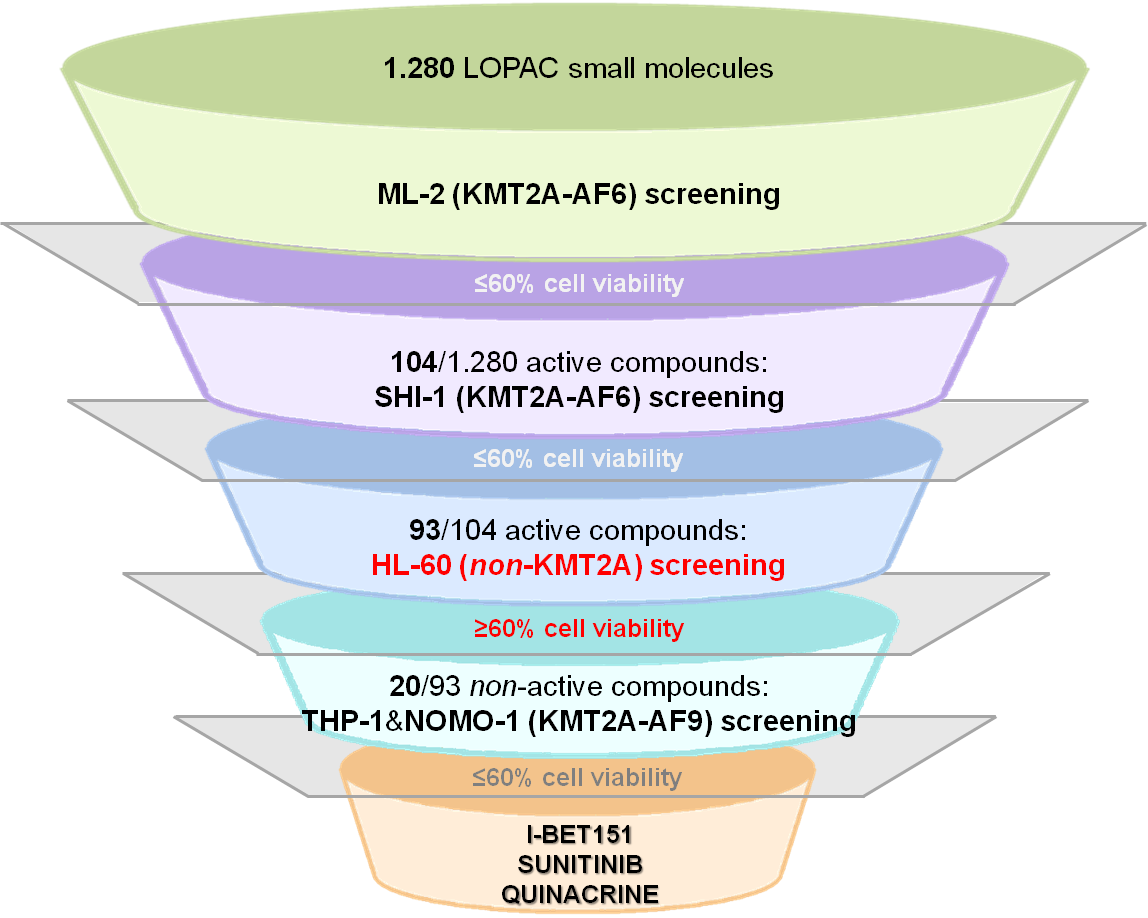


**Supplementary Figure 1**. Diagram showing the pipeline applied to the high throughput screening to identify drugs selective for KMT2A-rearranged AML.


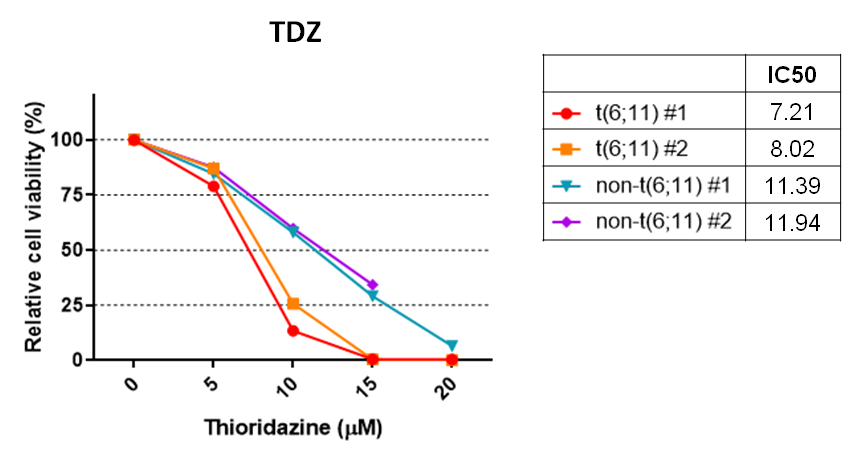


**Supplementary Figure 2.** Dose-response curve of growing concentrations of thioridazine in PDX-derived ex-vivo t(6;11) and non-t(6;11) AML, 48 hours after treatment (n=2).


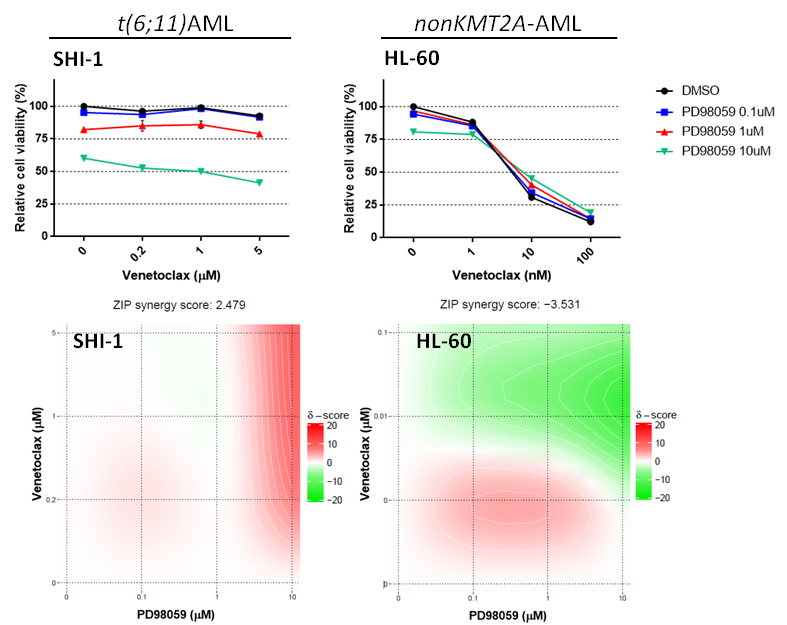


**Supplementary Figure 3.** Cell viability of t(6;11) SHI-1 and non-t(6;11) HL-60, after treatment with PD98059 combined with venetoclax, 48 hours after treatment. The synergy scores were represented by pseudocoloring 2-dimensional contour plots over the dose matrix (red indicates synergy and green indicates antagonism), and calculated using the ZIP model (synergy when >10, n=2).


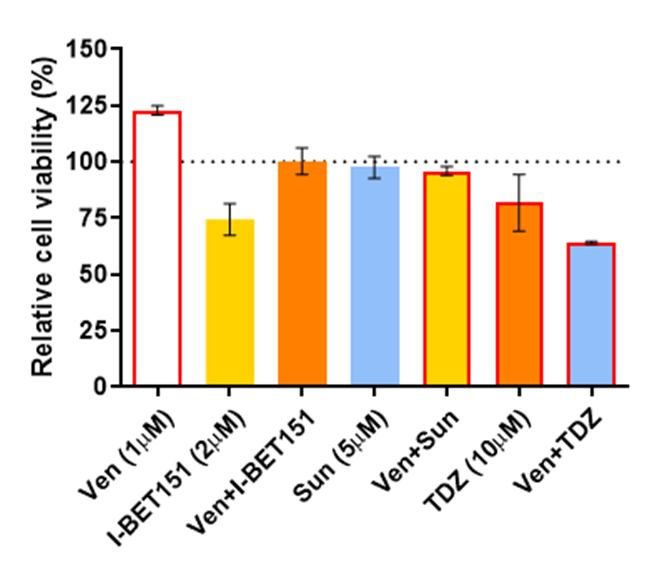


**Supplementary Figure 4.** Cell viability of 3D system seeded with AML-MSCs only, analyzed 48 hours after drug treatment with Venetoclax 1 µM, I-BET151 2 µM, sunitinib 5 µM and thioridazine 10 µM, normalized to the respective controls (DMSO) (n=3).

**
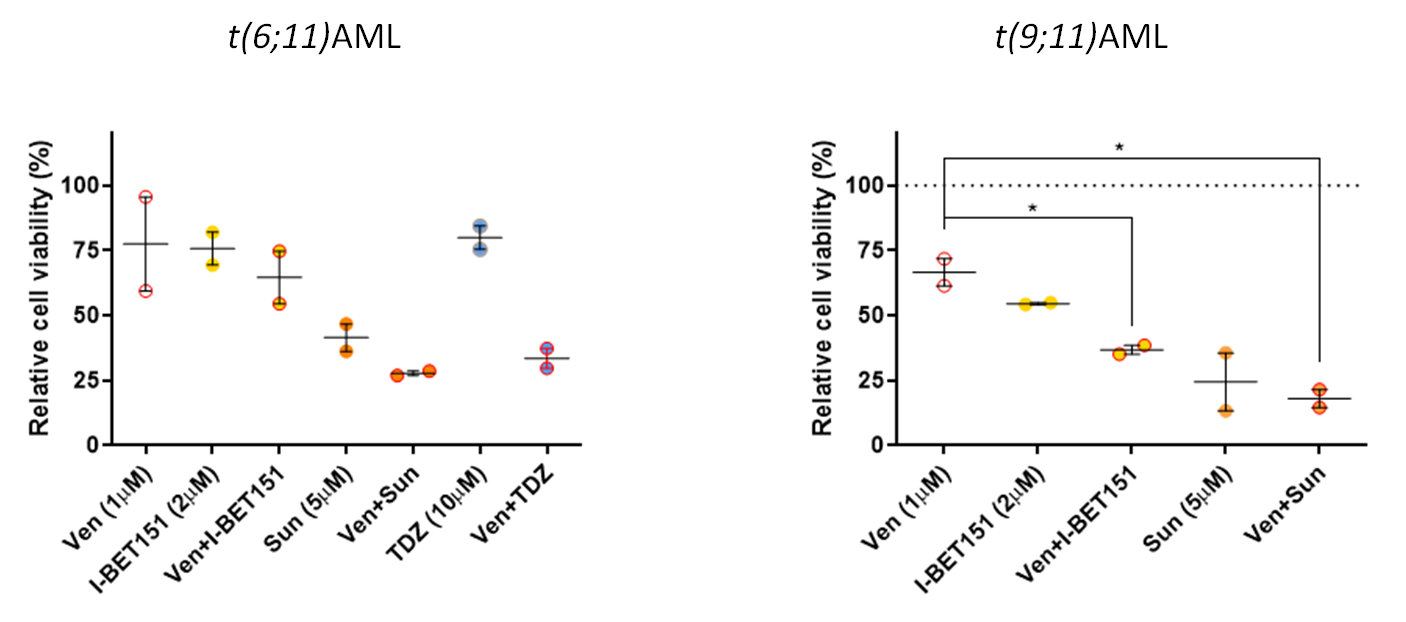
**

**Supplementary Figure 5.** Cell viability of 3D system analyzed 48 hours after drug treatment with Venetoclax 1 µM, I-BET151 2 µM, sunitinib 5 µM and thioridazine 10 µM, normalized to the respective controls (DMSO) (n=3) in AML primary samples. ANOVA test was performed applying Bonferroni correction for multiple statistical hypotheses testing. * p<0.05.
